# Characterization of Lymph Node Tumor Burden in Node-Positive Prostate Cancer Patients after Robotic-Assisted Radical Prostatectomy with Extended Pelvic Lymph Node Dissection

**DOI:** 10.3390/cancers15143707

**Published:** 2023-07-21

**Authors:** Josh Gottlieb, Shu-Ching Chang, Jane Choe, Gary L. Grunkemeier, Douglas A. Hanes, David Krasne, Dave S. B. Hoon, Timothy G. Wilson

**Affiliations:** 1 Department of Urologic Oncology, Providence St. John’s Cancer Institute, Santa Monica, CA 90404, USA; 2 Department of Biostatistics, Providence St. Joseph Health, Portland, OR 97213, USA; 3 Department of Pathology, Providence St. John’s Cancer Institute, Santa Monica, CA 90404, USA; 4 Department of Translational Molecular Medicine, Providence St. John’s Cancer Institute, Santa Monica, CA 90404, USA

**Keywords:** prostate cancer, prostatectomy, pelvic lymph node dissection, biochemical recurrence-free survival, BCR, lymph node metastasis

## Abstract

**Simple Summary:**

Prostate cancer (PCa) nodal staging does not account for tumor burden within the lymph nodes (LNs). In this retrospective single institution/single surgeon study, we assessed the significance of LN tumor burden in pN1 PCa patients after undergoing robotic-assisted radical prostatectomy with extended pelvic lymph node dissection. Consistent with prior reports, LN tumor burden was found to be significantly associated with biochemical recurrence-free survival (BRFS). This study was the first to report on the significance of the anatomical location of tumor deposits within the LN, as well as the quantified extent of extranodal extension (ENE). Likely due to sample size, the anatomical location within the LN and ENE did not show significant association with BRFS. We emphasize that PCa nodal staging and/or post-operative clinical nomograms should account for LN tumor burden.

**Abstract:**

Background: Prostate cancer (PCa) nodal staging does not account for lymph node (LN) tumor burden. The LN anatomical compartment involved with the tumor or the quantified extent of extranodal extension (ENE) have not yet been studied in relation to biochemical recurrence-free survival (BRFS). Methods: Histopathological slides of 66 pN1 PCa patients who underwent extended pelvic lymph node dissection were reviewed. We recorded metrics to quantify LN tumor burden. We also characterized the LN anatomical compartments involved and quantified the extent of ENE. Results: The median follow-up time was 38 months. The median number of total LNs obtained per patient was 30 (IQR 23–37). In the risk-adjusted cox regression model, the following variables were associated with BRFS: mean size of the largest LN deposit per patient (log2: adjusted hazard ratio (aHR) = 1.91, *p* < 0.001), the mean total span of all LN deposits per patient (2.07, *p* < 0.001), and the mean percent surface area of the LN involved with the tumor (1.58, *p* < 0.001). There was no significant BRFS association for the LN anatomical compartment or the quantified extent of ENE. Conclusion: LN tumor burden is associated with BRFS. The LN anatomical compartments and the quantified extent of ENE did not show significant association with BRFS.

## 1. Introduction

In 2020, there was an estimate of 1.4 million new prostate cancer (PCa) diagnoses and 375,000 deaths among men worldwide, making PCa the second most frequently diagnosed and fifth leading cause of cancer mortality worldwide [1]. For many years, standard treatments have consisted of radiation therapy, hormone therapy, and radical prostatectomy with or without pelvic lymph node dissection [2]. Performance of pelvic lymph node dissection (PLND) at the time of radical prostatectomy (RP) for PCa has been an extensively studied and controversial topic. PLND is performed on patients with intermediate or high-risk PCa when pre-operative nomograms indicate an estimated percent chance of lymph node involvement above 5–7%, depending on the nomogram utilized [3]. Despite the controversy in regard to survival benefits, the accepted rationale for performing PLND is that it provides improved staging and prognostic information that can help determine post-operative management. 

Pathological nodal status is important for risk stratification and clinical management in PCa. Current National Comprehensive Cancer Network (NCCN) guidelines provide a category 1 recommendation to start adjuvant androgen deprivation therapy (ADT) with or without external beam radiation therapy (EBRT) for lymph node involvement (LNI) after PLND [4]. However, the guidelines also list prostate-specific antigen (PSA) monitoring with early treatment for detectable and rising PSA or PSA >0.1 ng/mL as an option. While the discussion on adjuvant versus salvage therapy is not the goal of this paper, the essential point is that multiple management strategies exist based on the pathological nodal staging (PNS). 

The classification system for PCa nodal staging is surprisingly rudimentary in comparison to those developed for certain other solid malignancies such as breast, colon, and melanoma. Breast cancer PNS distinguishes between micrometastases and macrometastases, and also considers the size of metastasis and number of involved axillary LNs [5]. PCa nodal staging simply considers all patients with any amount of LN tumor burden to have pathologically confirmed LNI (pN1). 

Multiple studies already support an association of LN tumor burden with worse RFS/DSS/OS [6,7,8,9,10,11,12,13,14,15,16]. As early as 2000, Cheng et al. found that LN tumor volume was predictive of distant metastasis-free survival (MFS) and cancer-specific survival (CSS) [7]. In 2008, Fleischmann et al. prospectively showed that the diameter of the largest LN metastasis per patient was significantly associated with worse BCR-free survival (BRFS), CSS, and overall survival (OS) in patients with pN1 disease [9]. They subsequently found that stratifying pN1 patients by diameter of the largest LN metastasis (≤10 mm versus >10 mm) was the strongest predictor of recurrence-free survival (RFS), disease-specific survival (DSS), and OS. Deposits of >10 mm showed a quadrupled risk for BCR, and patients with “micrometastases” (defined as 0.2–2.0 mm) had the best survival outcomes [14]. With this data, they claimed that the TNM classification for nodal staging was unsatisfactory and that a sub-staging category should be created using a LN metastasis diameter of >10 mm vs. ≤10 mm. 

An increased number of involved LNs have also been shown to be independent predictors of decreased CSS and OS in prostate cancer [8,10,15,16]. The concept of updating the nodal staging system was reinforced by Briganti et al. in 2009 where they found that having >2 positive LNs was associated with worse CSS [16]. Passoni et al. compared the predictive ability of LN density (LND) and number of positive LNs on CSS and found similar discriminative powers between both these features. A cutoff of 30% LND was suggested to help determine which patients should receive adjuvant systemic therapy [13].

Despite all of this evidence, the PNS system has not been updated. As a result, all pN1 patients with varying extents of LN tumor burdens are subjected to the same post-operative recommendations and therapies. It seems counterintuitive that a patient with micrometastases in one involved LN should receive the same post-operative therapies as a patient with extensive LN metastases. With this in mind, the goal should be to stratify pN1 disease to determine which patients would benefit from adjuvant or salvage therapies. In order to do so, the amount of tumor burden within the LNs needs to be characterized with greater detail in hopes of developing an updated and clinically meaningful nodal staging system. 

To our knowledge, no studies have reported on the anatomical location of tumor deposits within the LN or the quantified extent of ENE. Our study aimed to characterize and assess pN1 disease with the greatest detail to date by analyzing the anatomical location of tumor deposits within the LN itself and the quantified extent of ENE, while also obtaining multiple other measurements of LN tumor burden. We hypothesized that our data would support the existing literature where greater LN tumor burden would be associated with reduced BRFS. We also hypothesized that the increased extent of ENE and LN capsular lymphatic involvement would be associated with reduced BRFS. 

## 2. Materials and Methods

### 2.1. Data Acquisition

This study was performed in accordance with the institutional review board protocol of Providence St. John’s Health Center (PSJHC). Clinical and pathological information was retrospectively collected via electronic medical record (EMR) from 66 pN1 patients after robotic-assisted radical prostatectomy (RARP) with ePLND was performed by a single surgeon from May 2015 to August 2021. All patients underwent ePLND as opposed to limited PLND due to the standard practice of the surgeon. Lymph node packets obtained from the right and left ePLND were the following: common iliac, external iliac, internal iliac, obturator, and node of Cloquet. Included patients could not have received any hormonal or radiation therapy prior to surgery. Hematoxylin and eosin (H&E) slides of all positive LNs were re-evaluated by a single pathologist. Clinical and pathological data were collectively arranged on both a “per patient” and “per lymph node” basis. Outcomes data were obtained from the PSJHC EMR, which included available records from outside institutions. Post-operative PSA values used for this study were from the time of 1st post-operative PSA (approximately 6 weeks after surgery), time of BCR, and most recent follow-up. Disease status at most recent follow-up was determined by most recent PSA (e.g., PSA < 0.2 ng/mL = patient without disease; PSA ≥ 0.2 ng/mL = disease present).

### 2.2. Pathological Characterization

For each patient, we recorded the number of total LNs obtained during ePLND as well as the number of positive LNs. For each positive LN, we recorded the LN packet where the positive LN was identified, the size of the largest tumor deposit within the LN, the total span of all discontiguous tumor deposits within the LN, the percent surface area (%SA) of the LN involved by tumor, the LN anatomical compartments involved, the presence of ENE, the span of ENE (mm), and the distance of ENE from the LN capsule (mm).

**Figure 1:**
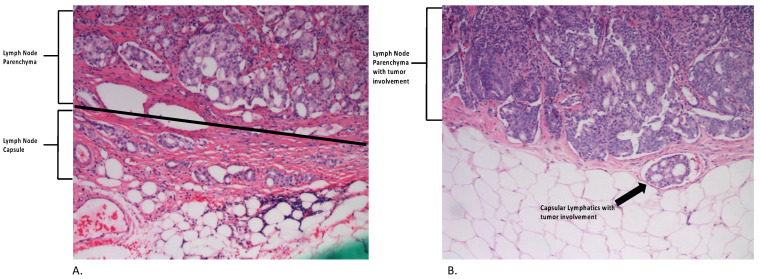
Lymph node anatomical compartments. (**A**) Above the line represents LN parenchyma. Below the line shows LN capsule thickened by tumor and associated fibrosis. (**B**) LN parenchyma involved with tumor. Arrow points to an endothelial-lined intracapsular lymphatic space containing tumor, which represents capsular lymphatic involvement. Both A and B are 20× magnification.

The LN anatomical compartments were subdivided into parenchyma, capsule, and capsular lymphatics (endothelial-lined intracapsular or pericapsular lymphatic spaces) (Figure 1). 

**Figure 2:**
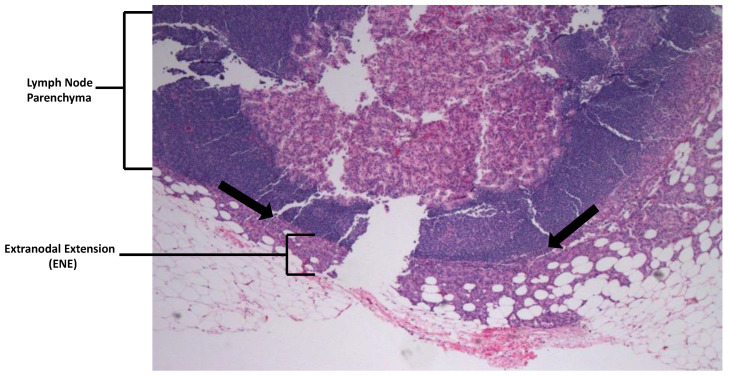
Lymph node extranodal extension (ENE). Arrows are at and parallel to a thinned LN capsule, with the parenchymal compartment above the arrows. Below the arrows, tumor is seen invading into perinodal fat qualifying as ENE. ENE was quantified in two dimensions: distance along the capsule (span of ENE), and distance from the LN capsule out into surrounding fat (distance of ENE from capsule). 10× magnification.

ENE was quantified in two dimensions: distance of the tumor along the LN capsule (span of ENE), and distance of the tumor from the capsule out into the surrounding adipose tissue (Figure 2). 

### 2.3. Study End Points

The primary outcome measure was BRFS, defined by a post-operative PSA of ≥0.2 ng/mL. Secondary outcomes were the need for adjuvant or salvage therapy (ADT and/or EBRT), and predictors of LN involvement. 

### 2.4. Statistical Analysis

Poisson regression analyses were performed to evaluate for predictors of LN metastases. BRFS was compared using the Kaplan—Meier method with log-rank test, followed by Cox proportional-hazards regression. The 17 patients with persistent post-operative PSA were excluded from this analysis because PSA persistence is known to be an independent risk factor for BRFS [17]. LN characteristics including the mean largest LN deposit per patient (mm), the mean total span of all LN deposits per patient (mm), the mean percent surface area of the LN involved with the tumor, the span of ENE (mm), and the distance of ENE from the capsule (mm) were based on continuous variables as well as dichotomized variables by median values for each variable. Statistical significance was defined as *p* < 0.05. All statistical analyses were performed using R software, version 4.1.2 (R Core team 2021).

## 3. Results

### 3.1. Patient-Level Clinical and Pathological Data

Patient-level clinical data are summarized in Table 1. A total of 66 pN1 patients after RARP + ePLND were included in the study. The median follow-up time was 38 months (IQR 26–50 months). A total of 17 out of 66 patients (26%) had detectable PSA (defined as PSA ≥ 0.2 ng/mL) on first post-operative PSA check. Of the 49 patients with undetectable PSA at first post-operative PSA check, 19 (39%) later developed BCR. Overall, 36 patients (55%) had either detectable post-operative PSA or BCR. A total of 38 patients (58%) received either adjuvant or salvage therapy with ADT and/or EBRT. At the most-recent follow-up, 48 patients (73%) were alive without disease, 14 patients (21%) were alive with disease, 3 (5%) were deceased without disease, and 1 patient (2%) was deceased with disease. Of the 48 patients without disease, 19 (40%) did not receive any form of salvage therapy

Patient-level pathological data are summarized in Table 1. The median post-surgery Grade Group was 5 (IQR 3–5). A total of 14 patients (21%) were staged as pT2, 19 (29%) as pT3a, and 33 (50%) as pT3b. 

A total of 51 (77%) patients had extraprostatic extension (EPE), 32 (48%) had seminal vesical involvement (SVI), 39 (59%) had lymphovascular invasion (LVI), and 53 (80%) had perineural invasion (PNI).

The median number of total LNs obtained from ePLND per patient was 30 (IQR 23–37). The median number of positive LNs per patient was 2 (IQR 1–3). The median size of the largest tumor deposit in all +LNs per patient was 2.6 mm (IQR 1.4–4.8 mm). The median span of all tumor deposits in all positive LNs per patient was 6 mm (IQR 2.5–13.5 mm).

### 3.2. Lymph Node-Level Pathological Data

Lymph node-level data are summarized in Table 2. A total of 148 LNs from the 66 patients were evaluated. The median size of the largest tumor deposit per positive LN was 2.5 mm (IQR 1.2–5 mm). The median span of all tumor deposits per positive LN was 3.5 mm (IQR 2–7 mm). The median percent surface area of positive LNs involved by the tumor was 8% (IQR 2–50%). A total of 144 (99%; n = 146) of the positive LNs had LN parenchyma involvement, 28 (19%; n = 144) had LN capsule involvement, and 27 (20%; n = 137) had LN capsular/pericapsular lymphatic involvement. A total of 46 (32%; n = 144) positive LNs had ENE. Of the LNs with ENE, the median span of ENE was 1.2 mm (IQR 0.8–3 mm), and the median distance of ENE from LN capsule was 0.4 mm (0.2–0.7 mm). 

### 3.3. Statistical Analyses for Predictors of BRFS and Incidence of Lymph Node Involvement

Poisson regression analyses showed that higher pre-surgery PSA, clinical stage T3-T4 versus T1-2, EPE, SVI, and LVI were significantly associated with a higher incidence rate of nodal positivity (Table 3). 

**Figure 3:**
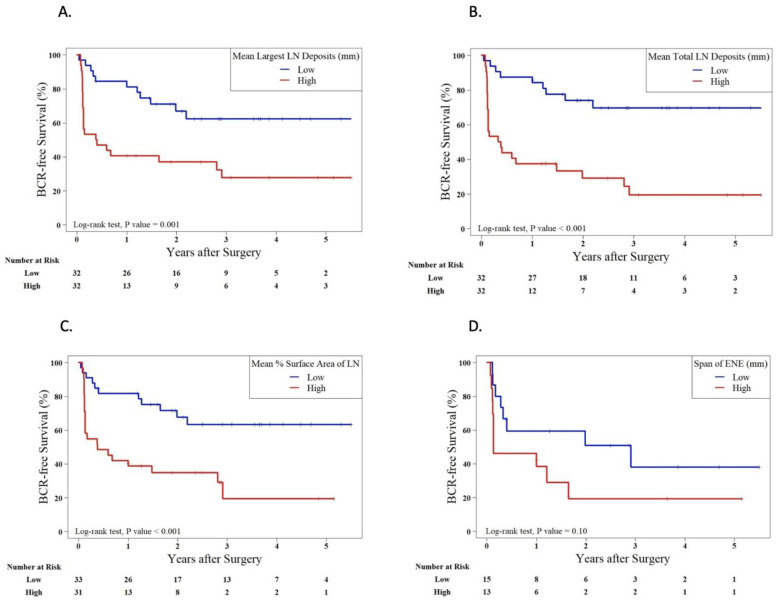

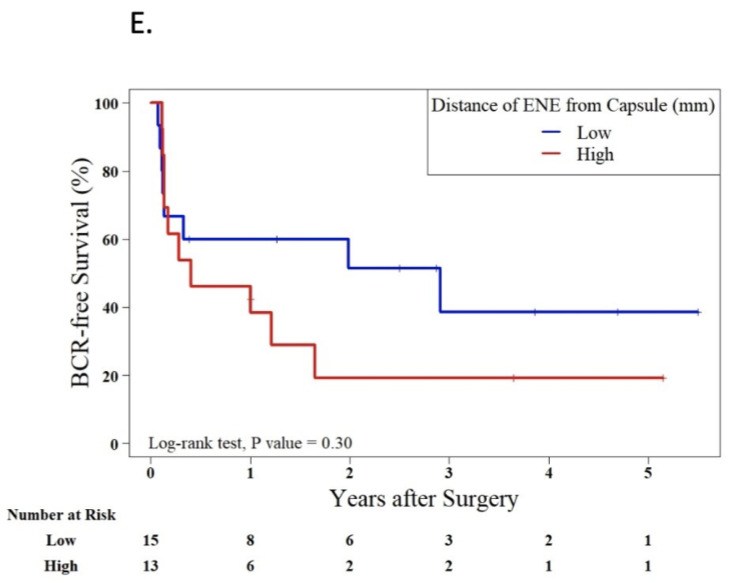
Kaplan–Meier survival curves stratified by median splits of (**A**) mean largest LN deposit per patient, (**B**) mean size of total LN deposits per patient, and (**C**) mean % surface area of LN involved with tumor. (**D**) Span of ENE and (**E**) Distance of ENE from LN Capsule.

**Figure 4:**
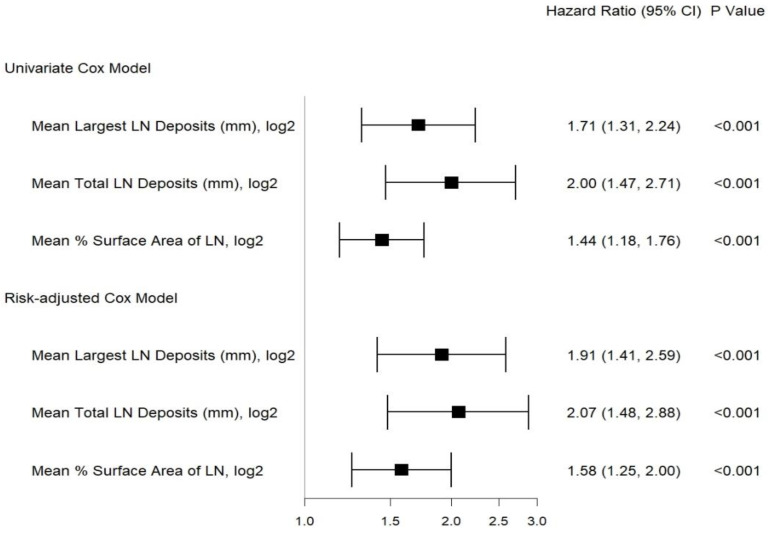
Forest plot showing univariate and risk-adjusted Cox regression analyses for the association of LN characteristics with BCR-free survival (adjusted for pre-surgery PSA, pathological stage, post-surgery Gleason grade, number of nodes examined, adjuvant therapy received, EPE, SVI, and LVI) at a median follow-up time of 38 months.

The median time from surgery to BCR was 17.6 (IQR 1.9–34.9) months. Kaplan–Meier analyses and univariate Cox regression showed that the mean size of the largest LN deposit, the mean total size of all LN deposits, and the mean percent surface area of LNs involved with the tumor were significantly associated with worse BRFS (Figure 3 and Figure 4). BRFS was not significantly associated with the number of positive LNs, LN parenchyma involvement, LN capsule involvement, LN capsular lymphatics involvement, ENE, span of ENE, and distance of ENE from capsule. Higher post-surgery Gleason grade (*p* = 0.03) and pre-surgery PSA (*p* = 0.01) were significantly associated with worse BRFS. 

Significant associations of the LN characteristics with BRFS remain after adjusting for age, pre-surgery PSA, clinical stage, post-surgery Gleason grade, number of nodes examined, EPE, SVI, and LVI (Figure 4).

## 4. Discussion

Consistent with prior reports, this study supports the association of LN tumor burden with worse BRFS in PCa. Specifically, our analysis found that the size of the largest LN deposit per patient, the total span of all LN deposits per patient, and the percent surface area of the LN involved with the tumor were all significantly associated with BRFS. The total number of LNs involved with the tumor was surprisingly not predictive of BRFS in this study. However, we believe this was due to the small sample size. Conceptually, “number of positive LNs” is a measure of LN tumor burden and was also found to be predictive of CSS and OS in other studies [8,10,15,16].

Our study adds to extensive research dedicated to LN tumor burden and how it relates to oncologic outcomes in PCa. The goal is risk-stratify pN1 disease to determine which of those patients would benefit from varying post-operative treatment modalities. In a randomized trial, Messing et al. showed an RFS rate of 16% in pN1 patients that did not receive secondary treatment after surgery [18]. Multiple other studies on the same patient population report RFS rates of approximately 11–28% at median follow-up times of up to 10 years [19,20,21]. This evidence shows that not all patients with pN1 disease require adjuvant or salvage therapy; thus, all pN1 patients should not be treated as a homogonous population. Yet, despite the long-standing evidence showing variable survival outcomes based on the extent of LN tumor burden, the nodal staging system has not been updated.

PSA persistence after definitive surgery is another important factor to consider in pN1 disease. In 2016, Bianchi et al. found that 26% of pN1 patients had PSA persistence (defined as PSA ≥ 0.1 ng/mL) after RP with ePLND at first PSA check. PSA persistence was found to be predictive of clinical recurrence and CSS, and having ≥3 positive LNs was predictive of PSA persistence. However, when analyzing the 74% of pN1 patients without PSA persistence, there was no survival or recurrence differences among those with ≥3 positive LNs versus <3 positive LNs [17]. This finding provided another potential method of stratifying patients with pN1 disease. Our study also reported 26% of patients with PSA persistence at first post-operative assessment. However, we defined PSA persistence as PSA ≥ 0.2 ng/mL due to our institutional practice of clinically observing PSA until ≥0.2 ng/mL (even in patients with pN1 disease). The main focus of our study was to characterize and analyze LN tumor burden, and so we did not assess for BRFS rates between PSA persistence and non-PSA persistence groups. We also did not assess for predictors of PSA persistence. 

Aside from updating the PNS system in PCa, post-operative clinical nomograms could be a useful approach to help risk-stratify pN1 patients and determine post-operative management. Ideally, these nomograms would determine when pN1 patients should receive adjuvant therapy or post-operative surveillance. Memorial Sloan Kettering Cancer Center (MSKCC) and Johns Hopkins University (JHU) have created two externally validated web-based post-operative nomograms for PCa that predict BCR probability up to 10 years after surgery [22,23,24]. Although the MSKCC nomogram accounts for nodal status, both nomograms do not account for LN tumor burden. In 2017, Nguyen et al. proposed an updated post-operative nomogram to predict BCR that stratified by number of positive LNs. They concluded that stratification by number of positive LNs improved the discriminative ability for evaluating BCR risk in LN-positive patients [25]. However, these nomograms are not routinely utilized on an international or national level. 

Without a clear set of clinical guidelines or validated nomograms accounting for LN tumor burden, patients with pN1 disease will continue to receive anecdotal and highly variable treatment algorithms in the post-operative setting. In addition, many patients with low volume LNI may continue to receive excessive and potentially unnecessary post-operative therapy [26,27]. For example, in 2021 Zattoni et al. reported that although adjuvant radiation therapy may improve recurrence-free survival compared to salvage therapy in patients with high risk for local recurrence, up to 40% of patients could be overtreated [28].

To the best of our knowledge, this is the first study that has assessed associations of LN anatomical compartment features and the extent of ENE with BRFS. Although unproven by this study, likely due to small sample size, these features still may be important. We believe these LN features should be further studied for potential inclusion in an updated nodal staging system or post-operative clinical nomogram. Another strength of this study is the lack of surgical bias by having all cases performed by a single surgeon with the same technique. Furthermore, all LN tissue slides were evaluated by a single pathologist to maintain consistency of histologic criteria and decrease interobserver variation. 

The primary limitations include the retrospective nature of this study, relatively small sample size, and short median follow-up time of approximately 3 years. However, most BCRs typically occur within 20–38 months after RP [29,30,31]. In addition, clinical outcomes were not stratified by those who received salvage or adjuvant therapy versus observation and by those with PSA persistence versus undetectable PSA post-operatively. Due to the small sample size with limited statistical power, further investigation with a larger cohort would be required to confirm the findings in this study.

## 5. Conclusions

Our data supports that LN tumor burden is associated with BRFS. In addition, our analysis found that higher pre-surgery PSA, pathological stage T3-T4 versus T1-2, EPE, SVI, and LVI were significantly associated with higher incidence rate of LN positivity.

Anatomical LN compartments and quantified extent of ENE did not show significant association with BRFS. This study further emphasizes the need to stratify pN1 PCa patients based on LN tumor burden, which could be in the form of an updated PNS system or post-operative clinical nomograms. 

## Figures and Tables

**Table 1:** Clinical and pathological characteristics of 66 included patients with pN1 PCa.

Patient-Level Clinical and Pathological Data	
Mean (SD) age at time of surgery	65.6 years (7.4 years)
Mean (SD) pre-surgery PSA	13.2 (12.3)
Number of patients with detectable 1st post-op PSA	17 (26%)
Number of patients with undetectable 1st post-op PSA	49 (74%)
Number of patients undetectable 1st post-op PSA that developed BCR	19 (39%)
Overall number of patients with detectable post-op PSA or BCR	36 (55%)
Number of patients that received any adjuvant/salvage therapy	38 (58%)
Number of patients that received adjuvant/salvage ADT	35 (53%)
Number of patients that received adjuvant/salvage RT	33 (50%)
Number of patients alive without disease at most recent follow-up	48 (73%)
Number of patients alive without disease + no adjuvant/salvage therapy	19 (40%)
Number of patients alive with disease at most recent follow-up	14 (21%)
Number of patients deceased without disease	3 (5%)
Number of patients deceased with disease	1 (2%)
Median (IQR) post-surgery Grade Group	5 (3–5)
Mean (SD) % volume of prostate involved with primary tumor	37% (23%)
Pathological Tumor Stage	
pT2	14 (21%)
pT3a	19 (29%)
pT3b	33 (50%)
Other Pathologic Features in Prostate	
EPE	51 (77%)
SVI	32 (48%)
LVI	39 (59%)
PNI	53 (80%)
Median (IQR) number of all LNs obtained in ePLND per patient	30 (23–37)
Median (IQR) number of +LNs per patient	2 (1–3)
Median (IQR) % of +LNs per patient	5.9% (3.6–9.1%)
Mean (SD) size of largest tumor deposit in all +LNs per patient	4.0 mm (4.5 mm)
Median (IQR) size of largest tumor deposit in all +LNs per patient	2.6 mm (1.4–4.8 mm)
Mean (SD) span of all tumor deposits in all +LNs per patient	12 mm (16.5 mm)
Median (IQR) span of all tumor deposits in all +LNs per patient	6 mm (2.5–13.5)

**Table 2:** Pathological characteristics of 148 LNs with tumor deposits collected from 66 patients with pN1 PCa.

LN-Level Pathological Data	
Mean (SD) size of largest tumor deposit per +LN	4.3 mm (5.1 mm)
Median (IQR) size of largest tumor deposit per +LN	2.5 mm (1.2–5 mm)
Mean (SD) total span of all tumor deposits per +LN	5.5 mm (5.4 mm)
Median (IQR) total span of all tumor deposits per +LN	3.5 mm (2–7 mm)
Mean (SD) % surface area of +LN involved by tumor	28% (34%)
Median (IQR) % surface area of +LN involved by tumor	8% (2–50%)
Number of +LNs with parenchyma involvement	144 (99%; n = 146)
Number of +LNs with capsule involvement	28 (19%; n = 144)
Number of +LNs with capsular lymphatics involvement	27 (20%; n = 137)
Number of +LNs with ENE	46 (32%; n = 144)
Mean (SD) span of ENE	2.1 mm (2.3 mm)
Median (IQR) span of ENE	1.2 mm (0.8–3 mm)
Mean (SD) distance of ENE from LN capsule	0.7 mm (0.9 mm)
Median (IQR) distance of ENE from LN capsule	0.4 mm (0.2–0.7 mm)

**Table 3:** Clinical and pathological risk factors for increased incidence of LN metastases.

Risk Factors for Increased Incidence of LN Metastases:	IRR	95% CI
Pre-surgery PSA, log2	1.31	1.15–1.50
Clinical stage T3-T4 vs. T1-2	1.92	1.27–2.90
Extraprostatic extension (EPE)	2.04	1.30–3.19
Seminal vesical involvement (SVI)	1.91	1.40–2.62
Lymphovascular invasion (LVI)	1.51	1.09–2.09

IRR = incidence rate ratio, CI = confidence interval.

## Data Availability

The data presented in this study are available on request from the corresponding author. The data are not publicly available due to ethical reasons.

## Abbreviations

ADT, androgen-deprivation therapy; aHR, adjusted hazard ratio; BCR, biochemical recurrence; BRFS, biochemical recurrence-free survival; CSS, cancer-specific survival; DSS, disease-specific survival; EMR, electronic medical record; ENE, extranodal extension; EPE, extraprostatic extension; ePLND, extended pelvic lymph node dissection; External beam radiation therapy, EBRT; IQR, interquartile range; IRR, incidence rate ratio; LN, lymph node; LNI, lymph node involvement; LVI, lymphovascular invasion; NCCN, national comprehensive cancer network; OS, overall survival; PCa, prostate cancer; PNS, pathological nodal staging; RARP, robotic-assisted radical prostatectomy; RFS, recurrence-free survival; RP, radical prostatectomy; RT, radiation therapy; SD, standard deviation; SVI, seminal vesicle involvement.
